# Clap-Trap Criticism

**Published:** 1890-10-11

**Authors:** 


### Passing Topics.

### CLAP-TRAP CRITICISM.

In our last issue we referred to the adjective "autocratic," which it has pleased some persons to apply to a certain lady whose name has been brought forward somewhat prominently of late. The condemnation of the matron of the London Hospital by these self-constituted judges has been arbitrary and complete, but, happily, judgment has not been pronounced without the opportunity being taken to point a moral.

ou btless the lesson lacks novelty, and we seem to have heard too often from similar sources of the general untrustworthiness of "paid officials" to feel very greatly disturbed by the pronouncement now. So far as the particular institution is concerned, we might be well content to balance these somewhat gratuitous philippics by a simple reference to testimony very much more to the purpose than the comments of journalistic outsiders in search of a means to quicken the torpid circulation of their precious prints—the unequivocal evidence of the Committee itself, that aggregation of unpaid elements, which, whatever its native shortcomings, is capable at any rate, one would suppose, of judging whether or no it is well and faithfully served.

We have already had occasion to deprecate the adoption by the matron of an independent attitude towards her nominal chief, and, maintaining our strictly impersonal standpoint, we will remark that neither the committee, the house governor, nor the matron appears altogether blameless in this matter. But though the very proper rule laid down had been obeyed with the utmost exactitude, none the less the actual direction of the hospital would still have remained in the hands of a paid officer, and we will join issue with our friends by asking—why not?

That it is undesirable the authority of any individual should be "autocratic" may be readily conceded. The word is not ours, and, as we venture to think, is used with only a partial appreciation of its real meaning. It is precisely because a paid officer is so easily held to his responsibility, and is therefore so essentially un-autocratic, that we prefer him to one who is unpaid. Wherever it can be shown that a sense of duty to a still higher authority has become impaired, the government of that institution is clearly faulty. It is a mere truism to assert that no person, or body of persons, ought to be in a position of complete irresponsibility in respect of the affairs they administer, and it may be reasonably contended that none can more nearly approach this position than the individual members of a body which acts collectively, and neither as a whole, nor in its constituent parts can be reached by any summary process, or visited with any serious consequences.

Good government in our hospitals is dependent upon a personal interest, for which there is no effective substitute. We should distinguish between control and management. Control by a committee, or its equivalent, is not only desirable, it is indispensable; but attempts at day by day management by committee must end in failure, for the obvious reason that the action and interest of a committee are by their nature intermittent, while vigilance and authority can never be safely relaxed.

Our amateur critics might well be asked how it is they fail to grasp a fact exhibited by society in all its grades—that the best and highest service is well paid for, and precisely as men attempt to perform continuous labour for nothing, is the quality of the work endangered. We do not shrink from the logical conclusion. The best committee would be a paid committee, every member of which could take his fee with the consciousness of having thoroughly earned it. With good and dutiful officers, the innovation may not be actually called for; but this is certain, that without them, a committee, whether paid or unpaid, would be hopelessly at fault.

Agricola.

